# Cryoballoon ablation of peri-mitral atrial flutter refractory to radiofrequency ablation: a case report

**DOI:** 10.1093/ehjcr/ytad598

**Published:** 2023-12-18

**Authors:** Masao Takahashi, Hirofumi Kujiraoka, Tomoyuki Arai, Rintaro Hojo, Seiji Fukamizu

**Affiliations:** Department of Cardiology, Tokyo Metropolitan Hiroo Hospital, 2-34-10 Ebisu Shibuya-ku, Tokyo 150-0013, Japan; Department of Cardiology, Tokyo Metropolitan Hiroo Hospital, 2-34-10 Ebisu Shibuya-ku, Tokyo 150-0013, Japan; Department of Cardiology, Tokyo Metropolitan Hiroo Hospital, 2-34-10 Ebisu Shibuya-ku, Tokyo 150-0013, Japan; Department of Cardiology, Tokyo Metropolitan Hiroo Hospital, 2-34-10 Ebisu Shibuya-ku, Tokyo 150-0013, Japan; Department of Cardiology, Tokyo Metropolitan Hiroo Hospital, 2-34-10 Ebisu Shibuya-ku, Tokyo 150-0013, Japan

**Keywords:** Case report, Cryoballoon ablation, Peri-mitral atrial flutter, Radiofrequency ablation, Epicardial conduction, Intramural conduction

## Abstract

**Background:**

The radiofrequency catheter ablation of peri-mitral atrial flutter is occasionally difficult, mostly due to epicardial or intramural conduction on the mitral isthmus (MI). However, cryoballoon ablation (CBA) of peri-mitral atrial flutter refractory to radiofrequency ablation has not been reported.

**Case summary:**

We report a case of a 66-year-old male patient who experienced a recurrence of atypical atrial flutter and underwent the sixth catheter ablation. The activation and entrainment maps showed that this atypical atrial flutter (AFL) was peri-mitral AFL via pathways other than endocardial conduction in the MI. Previous radiofrequency catheter ablation attempts on the MI line, including endocardial, coronary sinus, and epicardial ablations, failed to achieve a bidirectional block of the MI. In this case, we selected CBA for the MI area and successfully achieved a bidirectional block of the MI.

**Discussion:**

Although using CBA in the MI is off-label, it could be safely implemented using CARTOUNIVU™. We attributed the success of the bidirectional block of the MI in this case to the crimping of the northern hemisphere of the CBA to the mitral isthmus area, which resulted in the formation of a broad, uniform, and deep ablation lesion site.

**Learning points**

- Peri-mitral atrial flutter (AFL) is occasionally refractory to radiofrequency due to intramural or epicardial conduction.
- Although cryoballoon ablation (CBA) to mitral isthmus (MI) area is still an off-label treatment, it could be safely implemented with CARTOUNIVU™ in this case. CBA to MI area could form a broadly uniform and deep ablation lesion site in this case.

## Introduction

Previous reports have described peri-mitral atrial flutter (AFL) involving the epicardial or intramural layers in the tachycardia circuit.^1,2^ However, terminating AFLs using radiofrequency ablation is frequently difficult because of the mitral isthmus (MI) anatomical thickness and left pulmonary vein (LPV) ridge. We present a case where cryoballoon ablation (CBA) was useful for peri-mitral AFL refractory to radiofrequency ablation.

## Summary figure

**Figure:**
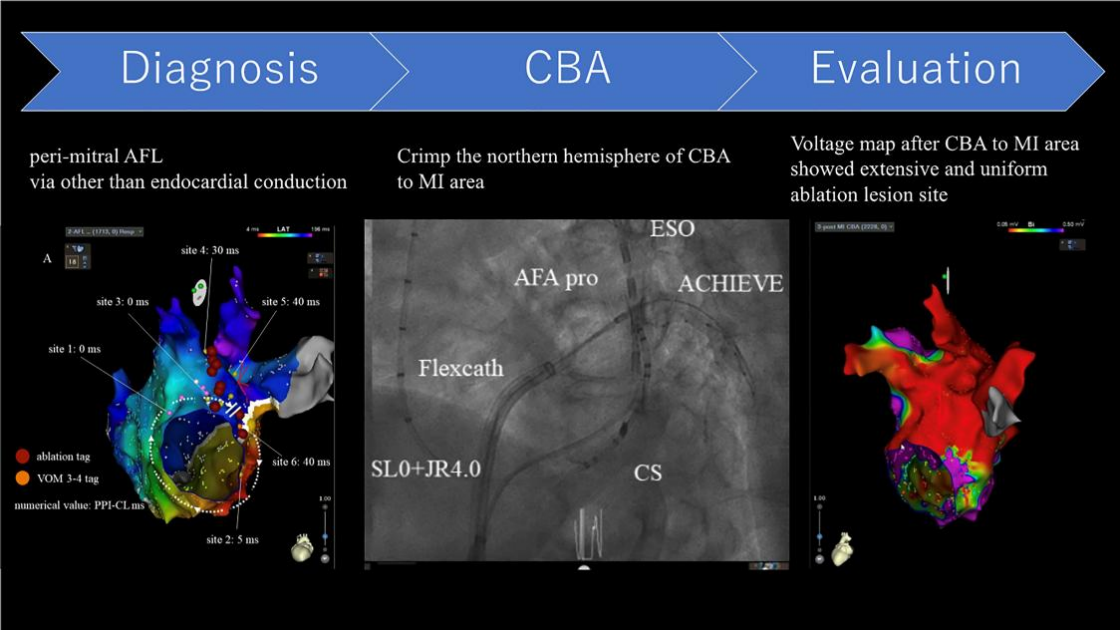


## Case presentation

**Figure 1:**
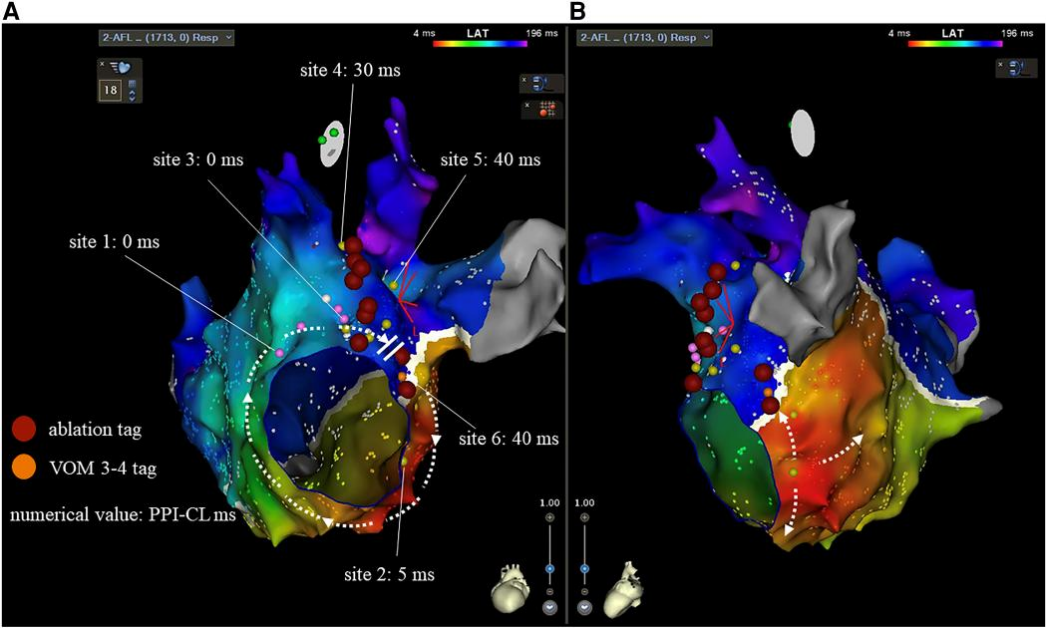
Activation and entrainment maps of atrial flutter of our case in left atrium. (*A*) Activation map showing the peri-mitral atrial flutter pattern, with a conduction gap in the mitral isthmus. Local activation time was only 200 ms of 265 ms (75%). Post-pacing interval-cycle lengths indicated 0, 5, 0, 30, 40, and 40 ms at the 12 o’clock position of mitral valve annulus (site 1), 4 o’clock position of mitral valve annulus (site 2), 2 o’clock position of mitral valve annulus (site 3), left pulmonary vein upper ridge (site 4), left pulmonary vein lower ridge (site 5), and vein of marshall 3–4 electrode on the endocardial block line in mitral isthmus (tag: site 6), respectively. radiofrequency ablation was performed at the left pulmonary vein ridge, left atrial appendage base, and mitral isthmus, as indicated by the red ablation tag. (*B*) Activation map of the centrifugal pattern from the 4 o’clock position of the mitral valve annulus.

**Figure 2:**
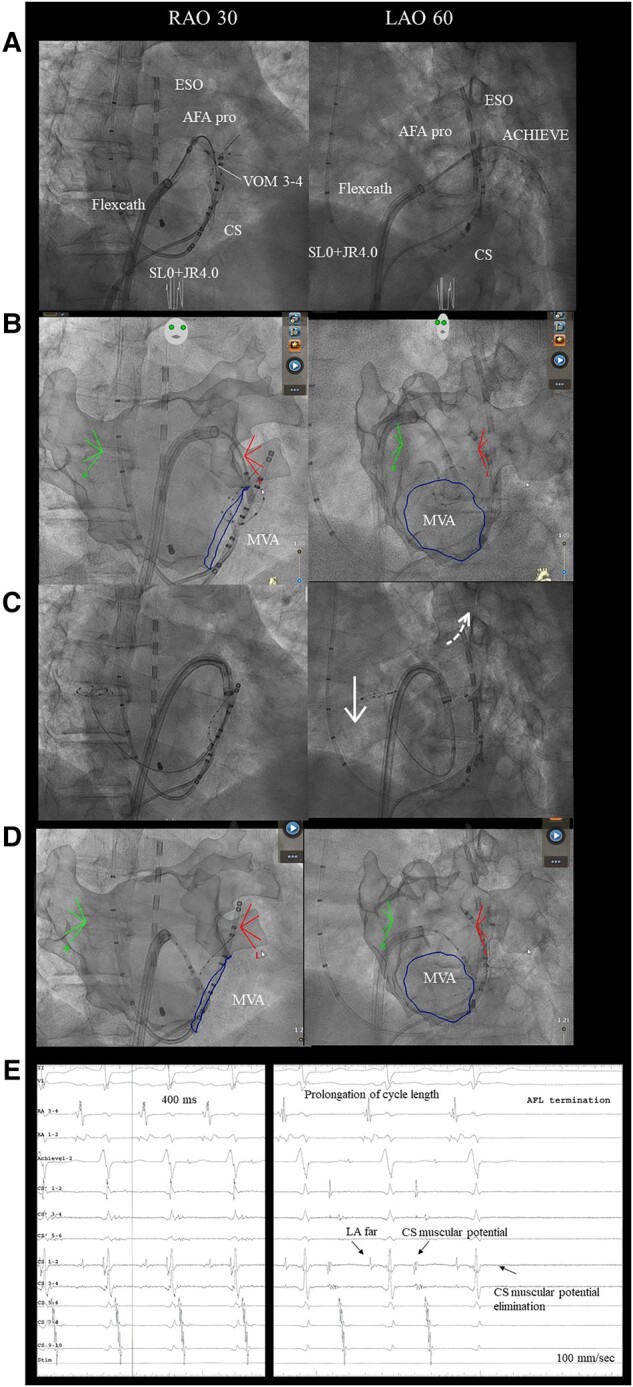
Cryoballoon ablation to the mitral isthmus and intracardiac electrocardiogram at the termination of the atrial flutter. (*A*) An eight-electrode mapping catheter is anchored into left inferior pulmonary vein, and the northern hemisphere of the cryoballoon is directed towards the mitral isthmus for ablation (freezing time: 180 s, minimum nadir balloon temperature: −38°C). (*B*) The steerable sheath is bent approximately 90° to the left atrial appendage base for the second ablation (freezing time: 180 s, minimum nadir temperature: −38°C). (*C*) The steerable sheath tip is bent downward, and the cryoballoon is gradually released. The sheath is subsequently unbent while pulling it downwards, and the northern hemisphere of the cryoballoon is directed towards the annulus side of the mitral isthmus. (*D*) The northern hemisphere of the cryoballoon is crimped to the annulus side of the mitral isthmus (freezing time: 180 s, minimum nadir balloon temperature: −37°C). (*E*) The left side shows an intracardiac electrocardiogram before the second cryoballoon ablation, and the cycle length is 400 ms. The right side shows an intracardiac electrocardiogram at the termination of atrial flutter during the second cryoballoon ablation to the left atrial appendage base.

**Figure 3:**
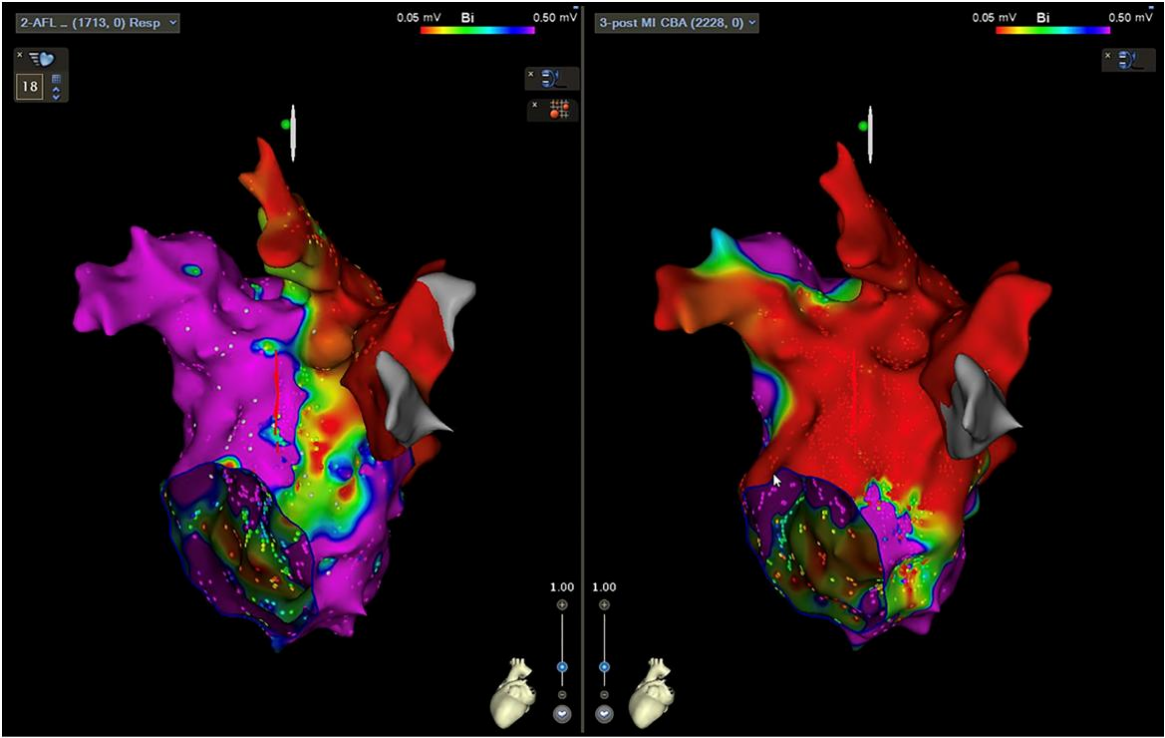
Pre-voltage and post-voltage maps. The left and right panels show the pre-voltage and post-voltage maps, respectively. In the post-voltage map, a homogeneous low-voltage area was extensively created in the mitral isthmus area.

A 66-year-old male patient underwent an electrophysiological study and five catheter ablation procedures for atrial fibrillation and atypical AFL. In the five previous catheter ablations due to radiofrequency, pulmonary vein (PV) isolation, cavo-tricuspid isthmus ablation, left atrial posterior wall isolation, atrial septum ablation, right PV carina ablation, superior vena cava isolation, and MI ablation, including coronary sinus (CS) and epicardial ablations, the MI line remained incomplete. During CS ablation, catheter impedance exceeded 200 ohms, and radiofrequency ablation was performed at a low output of 15–20 W. The patient complained of atypical AFL-induced palpitations that recurred 10 days after the fifth procedure, refractory to anti-arrhythmic drugs (bepridil and bisoprolol). The sixth catheter ablation for an atypical AFL was performed after obtaining written informed consent from the patient. He has no medical history other than this arrhythmia. On admission, the electrocardiogram showed 2:1 AFL with a heart rate of 124 bpm, echocardiography showed a left ventricular ejection fraction of 70% and left atrial diameter of 37.8 mm, and blood tests showed N-terminal pro-brain natriuretic peptide 446.9 pg/mL with no other special findings. The AFL activation map with a 265 ms cycle length in the left atrium (LA) was created using the CARTO 3 mapping system (Biosense Webster, Irvine, CA, USA) and a 20-electrode mapping catheter (PENTARAY; Biosense Webster) showing peri-mitral AFL-like colouration with clockwise rotation (see Supplementary material online, *Video S1*). However, the MI line indicated a conduction block on the endocardial side (*Figure 1A*) and a centrifugal pattern on the 4 o’clock of the mitral valve annulus (MVA) (*Figure 1B*). The local activation time was only 200 ms, suggesting epicardial or intramural crosslinking in the MI area. A 1.8-Fr catheter (EP Star, Lifeline, Tokyo, Japan) was inserted into the vein of marshall (VOM) using the femoral approach, and an entrainment map was created at various locations as follows: The post-pacing interval-cycle length (PPI-CL) values were 0, 5, 0, 30, 40, and 40 ms at the 12 o’clock position of the MVA, 4 o’clock position of the MVA, 2 o’clock position of the MVA, LPV upper ridge, LPV lower ridge, and VOM 3–4 electrode on the endocardial block line in the MI, respectively (*Figure 1A*, site 1–6). Based on the above findings, we diagnosed peri-mitral AFL via pathways other than endocardial conduction on the MI. When radiofrequency energy (40 W, 30–60 s) was applied, as shown in *Figure 1A*, cycle length prolongation without a change in the conduction sequence was observed (from 265 to 400 ms), although the AFL did not terminate. Considering the potential change in the tachycardia circuit, entrainment pacing was repeated at the same sites; however, PPI-CL had similar values. We considered that radiofrequency application from the endocardial side could not reach the tachycardia circuit extending from the intramural region to the epicardium and decided to perform CBA in the same area. First, an eight-electrode mapping catheter (ACHIEVE ADVANCE mapping catheter, Medtronic, Dublin, Ireland) was anchored into the left inferior PV (*Figure 2A*), and the northern hemisphere of the cryoballoon (Arctic Front Advance pro, Medtronic) was directed towards the MI for ablation (freezing time: 180 s, minimum nadir balloon temperature: −38°C). Subsequently, the catheter was pulled to the balloon tip, and the steerable sheath (FlexCath Advance, Medtronic) was bent approximately 90° to the left atrial appendage base for second ablation (freezing time: 180 s, minimum nadir temperature: −38°C) (*Figure 2B*). Finally, the MI annulus side was targeted. Since the northern hemisphere of the cryoballoon could not target the MI annulus side using similar catheter orientation as in the first and second CBA procedures, a third CBA was performed by bending the steerable sheath tip down and pulling the sheath downward while unbending and crimping the northern hemisphere of the cryoballoon to the MI annulus side (freezing time: 180 s, minimum nadir balloon temperature: −37°C) (*Figure 2C* and *D*). Overall, five CBA procedures were performed at different angles. During the second CBA, the cycle length was prolonged and terminated (*Figure 2E*). No electrocardiographic ST changes were observed during the CBA. Differential pacing confirmed a bidirectional block of the MI line. Postmapping was performed, confirming wide and uniform lesion formation in MI area (*Figure 3*). The session was completed without complications. The patient was symptom-free after discharge from our hospital and maintained sinus rhythm for 12 months.

## Discussion

To the best of our knowledge, this is the first report of CBA to the MI for peri-mitral AFL refractory to radiofrequency ablation other than endocardial conduction. Our previous case series showed that radiofrequency ablation of the LPV ridge or CS-marshall bundle connections effectively terminates peri-mitral AFLs using marshall bundle epicardial connections.^1^ However, in this case, peri-mitral AFL occurred via conduction pathways other than the endocardium and marshall bundle; therefore, radiofrequency ablation of the LPV ridge, left atrial appendage, and MI area could not terminate the peri-mitral AFL. Alcohol infusion or CS ablation was not performed because the VOM diameter was small, the VOM was outside the tachycardia circuit, and the high catheter impedance in the CS at the fifth procedure. Vlachos et al. reported radiofrequency catheter ablation to LPV ridge and CS-marshall bundle connections, and chemical ablation into VOM was unable to terminate macro-reentrant AFLs using marshall bundle epicardial connections of approximately 7%.^2^ Radiofrequency ablation cannot modify the tachycardia circuits in some cases. Therefore, CBA was selected because it is expected to form deep lesions uniformly over a relatively larger area than radiofrequency catheter ablation. We believe this CBA characteristic can increase the possibility of the ablation area reaching tachycardia circuits beyond the endocardium. However, some problems were associated with CBA to the MI, including technical issues, the durability of CBA lesions, and CBA settings, such as freezing time and target minimum temperature. Therefore, we believe these technical issues could be reduced using the CARTOUNIVU™ Module of Biosense Webster, which seamlessly combines a fluoro image and CARTO 3 system map into a single view. It helps reduce fluoroscopy levels to as low as reasonably achievable and exposure for physicians, staff, and patients, and we can navigate confidently from an integrated view with a single X-ray image or cine sequence for continuous anatomical orientation. Here, fluoroscopy images were created using CARTOUNIVU™ during confirmation of the contrast of cryoballoon occlusion to the left inferior PV, and the three-dimensional imaging of the LA on cardiac computed tomography was matched in each direction to provide an anatomical index. Using this anatomical index, as shown in *Figure 2B* and *D*, the northern hemisphere of the cryoballoon was accurately crimped and ablated against the left atrial appendage base and MI area. The post-voltage map in *Figure 3* shows that the CBA area aligns with the expected area. Although no reports exist on the durability of CBA to the MI, CBA to the left atrial roof with a comparable myocardial thickness has been reported. Shigeta et al. demonstrated that CBA to the left atrial roof for a freezing time of 180 s could produce durable lesions in 74.5% of patients with persistent atrial fibrillation.^3^ When comparing myocardial thickness in autopsy hearts,^4^ the mean value for roof and MI was 1.1 ± 0.5 vs. 1.6 ± 0.5 mm, and MI was slightly thicker. Although a computer model, Arctic Front Advance Pro can achieve cellular non-variability at a 3 mm depth for a freezing time of 160 s by allowing the tissue temperature to reach −20°C.^5^ Therefore, the threshold of cellular non-variability was likely reached because the nadir balloon temperature was below −37°C in all CBA procedures, and the freezing time was 180 s. CBA to the MI may be useful in refractory cases with recurrent peri-mitral AFL via pathways other than endocardial conduction, and using CARTOUNIVU™ may ensure safety. However, this is a case report, and further studies on the usefulness of CBA to the MI are necessary. Finally, we discussed the possible complications of CBA to the MI as follows: the effect on the coronary artery, both occlusion and spasm; the risk of phrenic nerve injury, which we believe infrequently occurs in the normal course of the left phrenic nerve; and the potential for an eight-electrode mapping catheter to become stuck in the mitral valve. Therefore, maintaining the mapping catheter in the LA whenever feasible is important. We successfully performed CBA to the MI for peri-mitral AFL refractory to radiofrequency ablation via pathways other than endocardial conduction.

## Patient perspective

He hoped to cure the atypical AFL by the sixth catheter ablation procedure.

## Supplementary Material

ytad598_Supplementary_Data

## Data Availability

The datasets generated and/or analysed during the current study are available from the corresponding author on reasonable request.

## Lead author biography

Graduated from the University of Occupational and Environmental Health in 2009.

## References

[1] Hayashi T , Fukamizu S, Mitsuhashi T, Kitamura T, Aoyama Y, Hojo R, et al Peri-mitral atrial tachycardia using the marshall bundle epicardial connections. JACC Clin Electrophysiol 2016;2:27–35. 29766850 10.1016/j.jacep.2015.08.011

[2] Vlachos K , Denis A, Takigawa M, Kitamura T, Martin CA, Frontera A, et al The role of marshall bundle epicardial connections in atrial tachycardias after atrial fibrillation ablation. Heart Rhythm 2019;6:1341–1347. 10.1016/j.hrthm.2019.05.019 31125669

[3] Shigeta T , Okishige K, Nishimura T, Aoyagi H, Yoshida H, Nakamura R, et al Clinical investigation of the durability of the lesions created by left atrial linear ablation with a cryoballoon. J Cardiovasc Electrophysiol 2020;31:875–884. 32017303 10.1111/jce.14379

[4] Hall B , Jeevanantham V, Simon R, Filippone J, Vorobiof G, Daubert J. Variation in left atrial transmural wall thickness at sites commonly targeted for ablation of atrial fibrillation. J Interv Card Electrophysiol 2006;17:127–132. 17226084 10.1007/s10840-006-9052-2

[5] Getman MK , Wissner E, Ranjan R, Lalonde JP. Relationship between time-to-isolation and freeze duration: computational modeling of dosing for arctic front advance and arctic front advance pro cryoballoons. J Cardiovasc Electrophysiol 2019;30:2274–2282. 31502304 10.1111/jce.14150 PMC6899473

